# Impairment of tissue repair in pneumonia due to β-cell deficiency: role of endoplasmic reticulum stress in alveolar macrophages

**DOI:** 10.1186/s13104-019-4209-0

**Published:** 2019-03-22

**Authors:** Yoshiro Yamashita, Reiki Kuroki, Masahiro Takaki, Takeshi Tanaka, Masachika Senba, Konosuke Morimoto, Hideaki Amano

**Affiliations:** 1 Department of Clinical Medicine, Institute of Tropical Medicine, Nagasaki University, Sakamoto 1-12-4, Nagasaki, Nagasaki 852-8523 Japan; 2 Department of Pathology, Institute of Tropical Medicine, Nagasaki University, Sakamoto 1-12-4, Nagasaki, Nagasaki 852-8523 Japan

**Keywords:** Diabetes mellitus, Pneumonia, Hepatocyte growth factor, Endoplasmic reticulum stress

## Abstract

**Objective:**

Diabetes mellitus (DM) patients are susceptible to delayed resolution of pneumonia. However, the pathogenesis of the impaired tissue repair in inflamed lungs in diabetic patients is unknown. We evaluated phagocytosis of apoptotic cells (efferocytosis), hepatocyte growth factor (HGF) production in bronchoalveolar lavage fluid (BALF), and lung histology in the resolution phase following acute lung injury in streptozotocin (STZ)-induced β-cell-depleted hyperglycemic mice. We also investigated efferocytosis and HGF production by macrophages under β-cell depletion condition ex vivo.

**Results:**

In β-cell-depleted mice, efferocytosis was not significantly different from that in control mice; however, the concentration of HGF in BALF was decreased. In addition, diminished HGF production by alveolar macrophages and DNA synthesis in the alveolar epithelium was observed by immunohistochemistry. Ex vivo experiments confirmed that HGF production by macrophages was impaired under β-cell depletion probably because of endoplasmic reticulum stress.

**Electronic supplementary material:**

The online version of this article (10.1186/s13104-019-4209-0) contains supplementary material, which is available to authorized users.

## Introduction

Diabetes mellitus (DM) is an independent risk factor for serious infectious diseases, including respiratory tract infections [1]. Furthermore, diabetic patients are prone to chronic bacterial pneumonia and un-resolving pneumonia [2, 3].

The engulfment and removal of apoptotic cells by phagocytes, known as efferocytosis, is involved in the regulation of inflammatory responses and the maintenance of lung tissue regeneration [4, 5]. Efferocytosis is suggested to induce the increases of the production of growth factors to promote tissue repair [6, 7]. Hepatocyte growth factor (HGF) is one of the most important growth factors and has pleiotropic effects, including regenerative, protective, and angiogenic activities [6, 8, 9]. HGF is produced by alveolar macrophages (AMs) that phagocytosed apoptotic neutrophils among resolution phase of injured lung [6] and is considered to be a pivotal growth factor for bronchial epithelial cells and alveolar type II cells [10, 11].

The endoplasmic reticulum (ER), an intracellular organelle, is involved in the synthesis and folding of transmembrane proteins into their proper three-dimensional structures. When ER stress is detected by the three types of sensors on the ER membrane (PERK, IRE1α, and ATF 6), the unfolded protein response (UPR) is induced to protect cells [12]. Once the UPR becomes impaired, the cell becomes apoptotic. Under diabetic conditions, ER stress is provoked in monocytes [13]. Therefore, we hypothesized that ER stress is also induced in AMs under diabetic conditions, and leads to impaired efferocytosis and HGF production and subsequent delayed resolution of pneumonia.

## Main text

### Methods

#### Model of acute lung injury in diabetic mice

Experimental diabetes was induced in 5-week-old, specific pathogen-free, male Sic:ICR mice (commercial source: Charles River Agricultural Cooperative Association, Kanagawa, Japan) with a single intraperitoneal injection of streptozotocin (STZ; Sigma-Aldrich, St. Louis, MO, USA) [14] at 250 mg/kg of body weight in 0.1 M citrate buffer (pH 4.5). Control mice received an equal volume of citrate buffer. Blood glucose levels were measured with a Glutest Sensor (Sanwa Chemical Co., Nagoya, Japan) to confirm that STZ treatment successfully depleted β-cells. Confirmed blood glucose levels after 48 h from STZ injection were 403 ± 23.97 mg/dl. More than 250 mg/dl were considered hyperglycemic. Ten days after administration, mice were anesthetized with an intraperitoneal injection of sodium pentobarbital (60 mg/kg), and the trachea was cannulated with an 18G cannula. Then, lipopolysaccharide (LPS; 5.0 mg/kg body weight) (Sigma-Aldrich) was administered intratracheally. After the experiment, mice were euthanized using deep anesthesia.

#### BAL and phagocytosis assay

Bronchoalveolar lavage (BAL) was performed as previously described [6] in each group of five mice at 0, 3, 6, 12, 24, 36, and 48 h, and 3, 4, 5, and 7 days after LPS inoculation. To observe macrophage engulfment of apoptotic neutrophils, the cells were fixed to a glass slide with cytospins, and then myeloperoxidase (MPO) activity was examined with the DAB Substrate Kit (Sigma-Aldrich) to visualize ingested neutrophils [15].

#### ELISA for HGF

The concentration of murine HGF in bronchoalveolar lavage fluid (BALF) was determined as previously described [6]. Briefly, we measured using a commercial rat HGF sandwich enzyme-linked immunosorbent assay (ELISA) kit (Institute of Immunology, Tokyo, Japan). We had already ascertained its usability for murine HGF in previous experiments [6].

#### Immunohistochemistry

At predesignated time points, mice were euthanized, and lungs were harvested. Paraffin-embedded specimens of whole lungs were prepared as previously described [6]. The number of HGF-positive alveolar macrophages was qualitatively assessed by visual inspection.

Proliferating cells in the tissue sections were assessed by immunostaining for proliferating cell associated nuclear antigen (PCNA). The dewaxed sections were stained for PCNA using a polyclonal antibody against human PCNA (Abnova, Taipei, Taiwan).

One mouse and two mice from the control and LPS-treated groups, respectively, were prepared, and four fields per mouse were imaged at 400× for blind counting by visual inspection.

#### Phagocytosis assay and HGF measurement ex vivo

Aged neutrophils, suspended in 300 μl of DMEM (Wako Pure Chemical Co., Osaka, Japan), were placed on cultured AMs. Then, the un-ingested cells were washed away, and the AMs were stained for MPO. Aged neutrophils were obtained using a technique previously reported [6, 15]. Harvested fresh neutrophils from mouse peritoneal cavity were incubated overnight at 4 °C in Hanks solution (Wako Pure Chemical Co). Neutrophils aged by these techniques were routinely 95 to 97% viable and around 70% apoptotic [6].

The concentration of HGF in the culture supernatants was determined as stated above.

#### Real-time PCR analysis

RAW264.7 cells (mouse peritoneal macrophage cell line) were cultured at 1–2 × 10^6^ in serum-free DMEM (standard glucose: 1 g/dl) containing with 3.5% albumin, not containing with insulin, in humidified 5% CO_2_ at 37 °C. Total RNA was extracted with the RNeasy Mini kit (QIAGEN, Gaithersburg, MD, USA). The extracted mRNA was quantified by measuring the absorbance, and was used as a template for RT-PCR to synthesize cDNA by SuperScript III (Invitrogen, Carlsbad, CA, USA).

The synthesized cDNA was then used as a template for real-time PCR with SsoFast EvaGreen Supermix and Low ROX (BIO-RAD, Hercules, CA, USA) as the enzyme. The primers used for *CHOP*, *Bip*, spliced *XBP*-1, total *XBP*-1, and β-actin have been previously described [16]. The PCR program consisted of an initial denaturation step at 50 °C for 30 s, followed by 40 cycles of denaturation at 95 °C for 5 s and annealing at 53 °C for 30 s.

#### Determination of HGF concentration under tunicamycin stimulation

RAW264.7 cells were cultured in DMEM supplemented with 10% heat-inactivated fetal bovine serum (FBS), 100 μg/ml streptomycin, and 100 U/ml penicillin in 5% CO_2_ at 37 °C at 1 × 10^6^ per well for 20 h. Tunicamycin (TM; Sigma-Aldrich) was added to the DMEM at 1 μg/ml, and DMSO was added to the control medium at 10 μl/ml. Apoptotic neutrophils [6, 15] suspended in 300 μl of DMEM without FBS, were added to the cultured RAW264.7 cells in triplicate and incubated for 20 h at 37 °C in 5% CO_2_. Culture supernatants were collected and centrifuged at 150×*g* for 5 min at 4 °C. Clarified supernatants were used to measure HGF concentrations.

#### Statistical analysis

Data are presented as mean standard error of the mean (SE). The statistical significance of differences between groups was determined by the unpaired (two-tailed) t test. One-way ANOVA was used for comparing several groups. A P value less than 0.05 was considered statistically significant.

### Results

#### Kinetics of inflammatory cells in lung injury

**Fig. 1:**
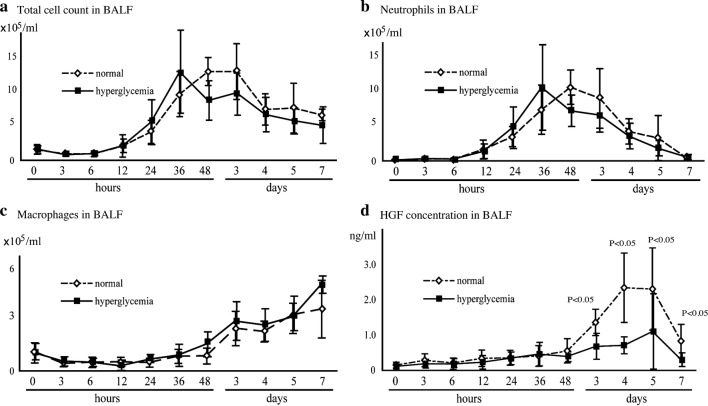
Kinetics of inflammatory cells and HGF production in LPS-induced lung injury in mice. **a** Time course of the total cells in BALF. **b** Time course of the neutrophil counts in BALF. **c** Time course of the macrophage counts in BALF. **d** HGF concentration in BALF as measured by ELISA. Open symbols represent control mice, and filled squares represent β-cell-depleted hyperglycemic mice. Data are presented as mean ± SE (n = 5). BALF from control mice contained 1.08 ± 0.38 × 10^5^ cells/ml, whereas BALF from β-cell-depleted hyperglycemic mice contained 1.19 ± 0.51 × 10^5^ cells/ml. There were no significant differences between control and β-cell-depleted hyperglycemic mice. HGF concentrations on days 3, 4, 5, and 7 were significantly lower in β-cell-depleted hyperglycemic mice than in control mice (P < 0.05)

We investigated the kinetics of cell infiltration into the airway following LPS-induced inflammation. The cell numbers of BALF were not significantly different between β-cell-depleted hyperglycemic and control (Fig. 1a). Analysis of the cells in BALF at each time point revealed that the increase in cell numbers was due to neutrophils, and there was a negligible change in the number of AMs (Fig. 1b, c).

Neutrophil influx into the lungs of control mice was apparent at 24 h. Then increased, and reached a peak at 48 h. In β-cell-depleted mice, neutrophil influx was also detected at 24 h, reached a peak at 36 h, and then gradually decreased over a 7-day period. These results show that in control mice, inflammation was initiated within 24 h and reached a peak at 36 h. In the β-cell-depleted mice, the numbers of neutrophils at each time point were not significantly different, and the peak level was equivalent (Fig. 1b).

#### Neutrophil removal and HGF production in lung injury

The number of MPO-positive macrophages began to increase at 24 h (7.0 ± 6.0% of total AMs) in control mice, reached a maximum at day 3 (29 ± 15% of total AMs), and remained high up to day 5. The kinetics of MPO-positive AMs were similar among the groups (Additional file 1: Figure S1A). The number of AMs remained unchanged from baseline until 36 h, but started to increase at 48 h (Fig. 1c). After 48 h, the percentage of neutrophils gradually decreased until the end of the observation period (Fig. 1b). Taken together, these findings indicate that the efferocytosis activity was augmented following LPS administration and contributed to the resolution of acute inflammation, especially between 48 h and 5 days.

The concentration of HGF in BALF reached a peak on day 5 in both groups; however, it was significantly lower in β-cell-depleted mice (1.656 ± 0.714 ng/ml) than in control mice (4.093 ± 0.693 ng/ml). Furthermore, the HGF concentrations in control mice were continuously higher than those in β-cell-depleted mice from day 3 to day 5 (P < 0.05, Fig. 1d).

#### HGF production and DNA synthesis in the lung were attenuated in β-cell-depleted hyperglycemic mice

**Fig. 2:**
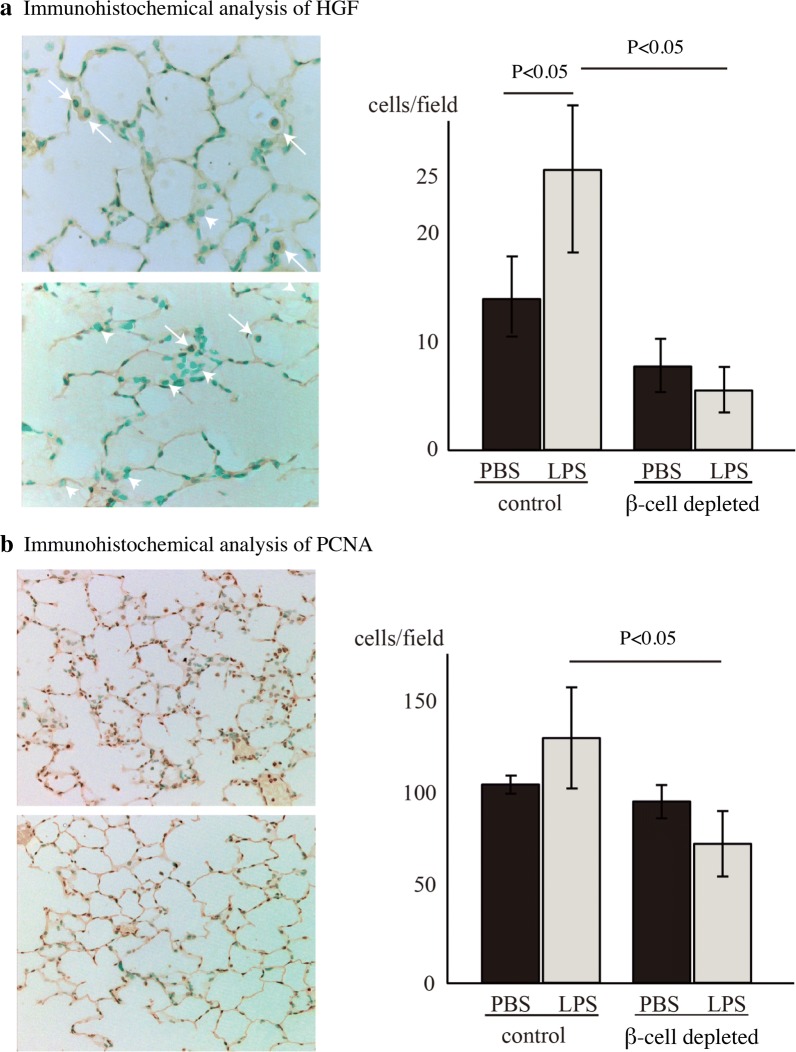
Immunohistochemical analysis in mouse lungs. **a** Representative image of immunohistochemical analysis of HGF. Arrows indicate HGF-positive alveolar macrophages (AMs). Arrow heads indicate HGF-negative AMs. Right side graph indicates positive cells per field. Data are presented as mean ± SE (n = 3). There were significant differences between PBS-treated and LPS-treated control mice, and between LPS-treated control and β-cell-depleted hyperglycemic mice (P < 0.05). **b** Typical image of immunohistochemical analysis using anti-PCNA polyclonal antibody. Upper picture, control mouse; lower picture, β-cell-depleted hyperglycemic mouse. Right side graph indicates that the numbers of positive cells per field were significantly different between LPS-treated control and β-cell-depleted hyperglycemic mice (n = 3, P < 0.05)

HGF-positive cells were present within bronchial epithelial cells and AMs at 5 days after LPS administration in both mouse groups (Fig. 2a). In β-cell-depleted mice, the number of HGF-positive AMs was much lower than that in control mice (P < 0.05, Fig. 2a).

To assess regenerative proliferation, cells undergoing DNA synthesis were identified by anti-PCNA polyclonal antibody. In both groups, the PCNA-positive cells were mainly alveolar epithelial cells. Consistent with the difference in HGF production, there were much fewer PCNA-positive alveolar epithelial cells in β-cell-depleted mice than in the control mice on day 5 (P < 0.05, Fig. 2b).

#### HGF production was suppressed in β-cell-depleted mouse macrophages

**Fig. 3:**
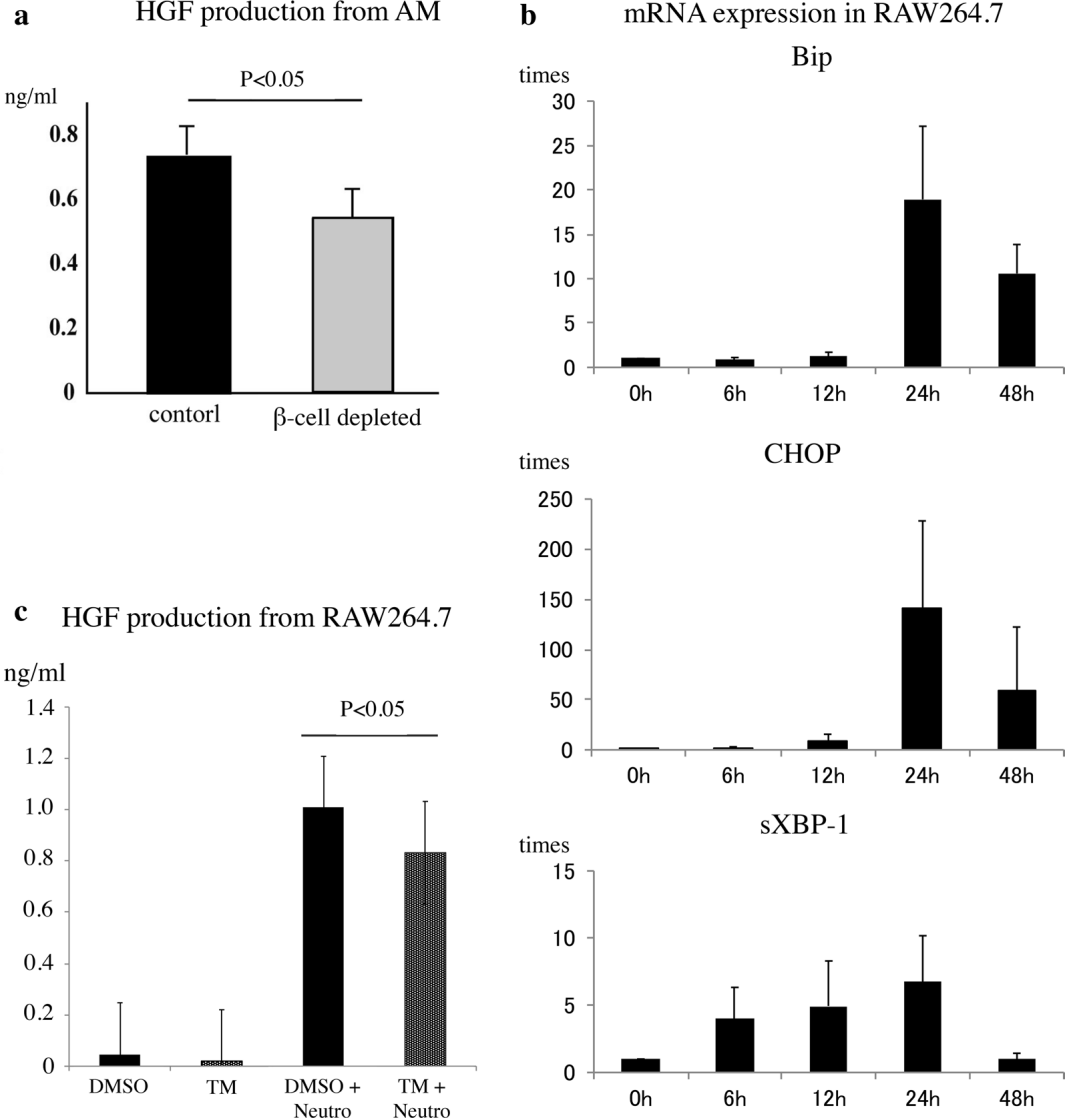
Ex vivo and vitro analysis for β-cell-deficiency and ER stress. **a** HGF concentration in culture supernatant measured by ELISA. Data are presented as mean ± SE (n = 3). There was a significant difference in HGF production between the control and β-cell-depleted hyperglycemic mice AMs (P < 0.05). **b** UPR mRNA expression in the RAW264.7 mouse macrophage cell line after insulin starvation. mRNA expression of UPR-related genes: *Bip*, *CHOP*, and *sXBP-1* evaluated by real-time PCR. mRNA expression was evaluated over time. The results are shown as ratios to the mRNA expression of β-actin and tXBP-1. Data are presented as mean ± SE (n = 3). The mRNA expression of the two UPR-related molecules (*Bip* and *CHOP*) was significantly increased after insulin starvation (P < 0.05). Also, the mRNA expression of *sXBP-1* was remarkably increased after insulin starvation. **c** HGF production by RAW264.7 macrophages in vitro under ER stress. RAW264.7 macrophages were treated with TM (1 μg/ml), and apoptotic neutrophils were added. Then, the HGF concentration in culture supernatant was measured by ELISA. Data are presented as mean ± SE (n = 3). There were significant differences in HGF production from RAW264.7 cells with apoptotic cells between cells treated with and without TM (1 μg/ml; P < 0.05), whereas apoptotic cells uptakes were comparable

We examined the efferocytosis of AMs and measured the concentrations of HGF in culture supernatants. The percentage of MPO-positive AMs, was similar in the two groups (Additional file 1: Figure S1B); however, HGF production was significantly lower in the AMs from β-cell-depleted mice than in the AMs from control mice (P < 0.05, Fig. 3a).

#### mRNA expression of UPR was increased in insulin-starved macrophages.

At first, we compared mRNA expression between culture with standard glucose (1 g/dl) medium and culture with high glucose (4.5 g/dl) medium. After 5 days culture, however we found no significant differences between those conditions. At this point, we thought of the influence of insulin starvation. Then, we performed a time course analysis under insulin starvation. After 24 h, the mRNA expression of *Bip* and *CHOP* suddenly reached peak levels, whereas the mRNA expression of *sXBP*-1 increased in an earlier phase (Fig. 3b). These results suggested that diabetes causes ER stress in macrophages under insulin-starved or insulin-resistant conditions.

#### HGF production was suppressed under ER stress.

ER stress may play a role in suppressing HGF production by mouse AMs. We tested this hypothesis by applying a strong ER-stress inducer, TM, to the cells. In the absence of apoptotic neutrophils, the RAW264.7 did not produce HGF; however, in the presence of apoptotic neutrophils, the macrophages produced HGF. When we added TM to the RAW264.7, the HGF concentration in the culture supernatant decreased (P < 0.05, Fig. 3c).

### Discussion

Our data improve our understanding of the pathogenesis of delayed-resolving pneumonia in diabetic patients. HGF production during the resolution phase of inflammation was impaired in β-cell-depleted mice. Following impaired tissue repair may subsequently lead to poorer outcomes for patients with pneumonia. The mechanism of suppressed HGF production is unknown; however, we could speculate that it might be due to acceleration of the PERK-eIF2-α-ATF4-CHOP pathway or inhibition of endoplasmic-related degradation (ERAD) in the UPR [17]. PI3K/AKT signal pathway is also suggested to regulate HGF expression [18, 19]. Considering the insulin signal that is mediated by PI3K/AKT pathway [20], we supposed that suppressed PI3K/AKT pathway activation due to insulin starvation additionally decreased HGF expression in this study.

Contrary to our hypothesis, ER stress had a negative effect on efferocytosis. The diabetic condition is characterized by hyperglycemia, acidemia, higher osmotic pressure, intracellular starvation, and ER stress. We postulate that these may have complicated effects on macrophage function. Further, adding to the differences between alveolar and peritoneal macrophages, in the resolution phase of lung inflammation, recruited macrophages uptake apoptotic cells more effectively [21].

## Limitations

First, the STZ-induced hyperglycemia model may more closely reflect Type1 diabetes because β-cell depletion is a feature of Type1 diabetes. Second, the blood sugar levels of the mice were very high, in the range of severe diabetes. Thus, our model does not accurately reflect common clinical diabetes. Third, our study included animals and ex vivo experiments; the alveolar macrophages in humans possibly express different features.

## Additional file

**Additional file 1: Figure S1**. Efferocytosis of mouse AMs. A) Percentage of MPO-positive AMs after intratracheal injection of LPS. Open symbols represent control mice, and filled squares represent β-cell-depleted hyperglycemic mice. Data are presented as mean ± SE (n = 5). There were no significant differences in MPO-positive AMs between the control and β-cell-depleted hyperglycemic mice. B) Percentage of MPO-positive AMs ex vivo. Data are presented as mean ± SE (n = 3). There were no significant differences in MPO-positive AMs between the control and β-cell-depleted hyperglycemic mice.

## Abbreviations

DM: diabetes mellitus
HGF: hepatocyte growth factor
AMs: alveolar macrophages
ER stress: endoplasmic reticulum stress
UPR: unfolded protein response
STZ: streptozotocin
LPS: lipopolysaccharide
MPO: myeloperoxidase
BALF: bronchoalveolar lavage fluid
PCNA: proliferating cell associated nuclear antigen
FBS: fetal bovine serum
TM: tunicamycin
