# Supplementary figures and images for: Impairment of tissue repair in pneumonia due to β-cell deficiency: role of endoplasmic reticulum stress in alveolar macrophages
Source: BMC Res Notes. 2019 Mar 22;12:160. doi: 10.1186/s13104-019-4209-0 (PMC6431046; doi:10.1186/s13104-019-4209-0)

## A Percentage of MPO positive AMs

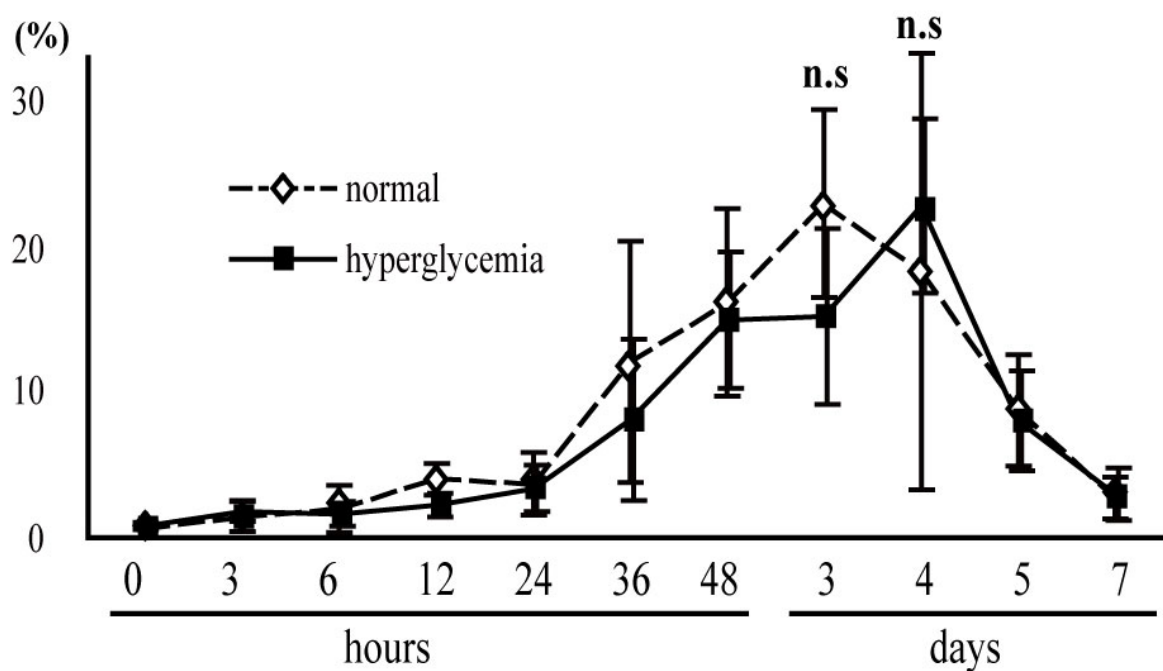

## B Percentage of MPO positive murine AMs

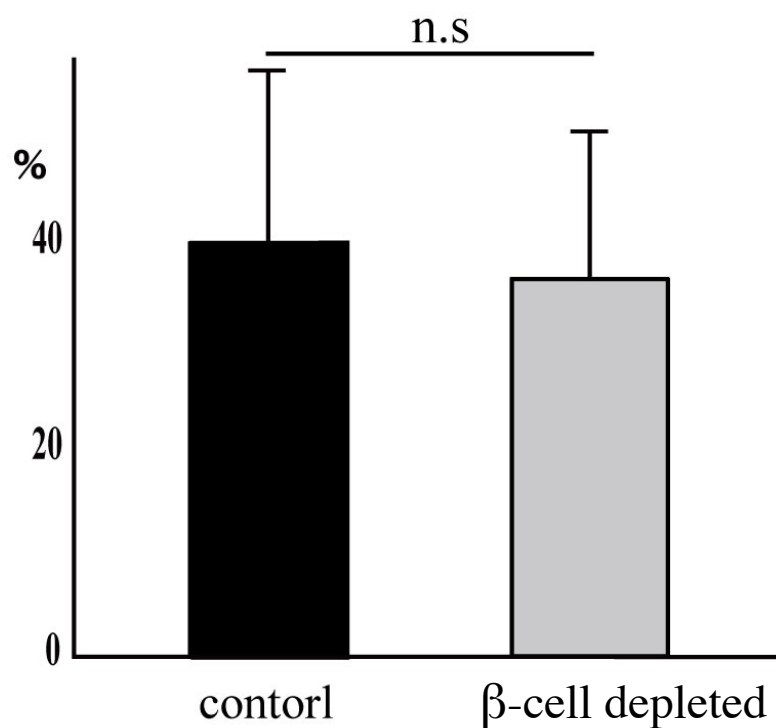

Supplement: Supplementary file 1 — Additional file 1: Figure S1. Efferocytosis of mouse AMs. A) Percentage of MPO-positive AMs after intratracheal injection of LPS. Open symbols represent control mice, and filled squares represent β-cell-depleted hyperglycemic mice. Data are presented as mean ± SE (n = 5). There were no significant differences in MPO-positive AMs between the control and β-cell-depleted hyperglycemic mice. B) Percentage of MPO-positive AMs ex vivo. Data are presented as mean ± SE (n = 3). There were no significant differences in MPO-positive AMs between the control and β-cell-depleted hyperglycemic mice. [file 13104_2019_4209_MOESM1_ESM.pdf]
